# Autobiography of the Insane

**Published:** 1856-01-01

**Authors:** 


*(To be continued.)*

---

### Art. V.—AUTOBIOGRAPHY OF THE INSANE.

[The writer of the following narrative was for some period an inmate of Bethlehem Hospital, under the kind and skilful care of Dr. Hood, the physician of that establishment. Dr. Hood requested the writer, after his recovery, to describe his state of mind and sensations during his attack of insanity. The details he has given will be read with deep interest by all engaged in psychological investigations, and in the treatment of the insane.—*Editor*.]

Agreeably to your wishes, I will undertake to make you acquainted with, 1st, the causes from which, in my opinion, originated the disease that has brought me into this establishment; 2nd, my various sensations and thoughts whilst I was labouring under it.

Previous to this year, 1851, I never for one moment suffered from mental derangement, although, I must confess, that I commenced to take strong drinks with excess, at a period so far back as the latter end of 1849; until that time the only complaint I was subject to was accidental constipation, accompanied by fever and loss of appetite.

As my disease first made its appearance in Londonderry, I

shall take the liberty of giving you an account of my way of living there, from the time of my arrival to the day when illness, despair, and want of pecuniary resources, compelled me to leave it.

In August, 1848, on my return from France, whither I had gone to spend my vacation, I was, on the most pressing recommendation of the Manager of the Bank at Larne, who knew me, appointed French master at Foyle College, Londonderry. The Reverend Mr. Henderson, who was and still is the head-master, after the trial of a few days to put my qualifications to the test, agreed with me that I should receive my board and lodging in the establishment, in return for French tuition imparted to a limited number of pupils: my lessons were to be given four times a week, and to last two hours every time.

For three months I lived in the college, attending my classes there according to the agreement, and also other young gentlemen and ladies in town; but finding that I could not meet every one's wishes without interfering with the meal-hours at college, I resolved on taking up my residence in the city. The principal, to whom I communicated my determination, gave me his full approval, and desired me to continue my attendance in his establishment for the salary of one pound per quarter for each pupil.

I therefore removed on the 1st of December and got lodgings in a most respectable family, consisting of four sisters. They were elderly ladies, and nearly related to a gentleman whose daughters I attended.

There I spent, until June, the most happy months I ever enjoyed. My health was excellent, I had as many scholars as I could wish; the ladies of the house were more like sisters than strangers to me, and the steadiness of my conduct as a teacher caused the best families in and about Derry to honour me with their esteem. In a word, I saw before me most encouraging prospects, but there was in me, steady, sober as I was, the seed of many sins, a profound disrespect for religion.

Like many of my countrymen, and though brought up by a most pious mother, I was a Christian only by name. The college life in Paris had almost rooted out from me all notion of God. Thus, whilst in the sight of men my conduct was irreproachable, I shamefully forgot that the discharge of our duties towards our Creator is alone calculated to render our conduct irreproachable. Never did I once go either to church or to chapel during upwards of two years.

I returned to France, as usual, in June 1849, and came back in August next, after a stay of a few weeks with my family and friends; but there too I was so obstinate in my refusing any attendance at church, on Sundays, that I left my poor mother quite dissatisfied at what she called my *déplorable esprit fort.* Many, many a time did she prophesy to me that I should one day weep on my impiety.

I was soon to experience the realization of that prediction. When I came back, I found on my arrival, a new servant occupied in my sitting room. She had been engaged, during my absence, to replace the elderly woman who used to wait on me at table, and to do whatever I might require. I was very much satisfied with her attendance, and sincerely regretted her discharge. On my asking why she had been dismissed, I was answered that she could not do all the work, and that a young, active girl was by far preferable.

The new servant was young indeed, and possessed of some attractions, which I was foolish and imprudent enough not to resist; but for my attention to which I have since been severely punished. Let it suffice to say that I yielded to temptation. From that time, I can assert it, may be traced all my troubles and misfortunes. The girl, though young, was knowing enough to perceive that I was in her power more than she was in mine. She openly told me so more than once. In the mean time, she took great care to obtain from me as much money as she could. I then commenced to drink whisky mixed with water, first in small quantity and only at night, after my business was over. The *libations* became by degrees more frequent and copious, especially when she apprised me that she was with child, and consequently expected that I should marry her.

I cannot describe to you, Monsieur le Docteur, the state into which that unpleasant news, expected as it might have been, threw my mind. I saw that my ruin was unavoidable, whatever plan I might adopt. If I do not marry her, said I, she will make a scandal, and I shall be obliged to leave the town. If on the other hand I marry her, I am sure to fall into discredit and to lose most of my pupils.

This happened at the latter end of March, 1850. Instead of returning to better sentiments, and praying to God that he would inspire me with the means of averting the catastrophe, by sending the girl out of town, with a sufficient maintenance, until I should be able to atone for my fault in the only honest way, that is, in marrying her, but so as to keep our marriage secret, I became the more reckless of the time to come, went on drinking whisky, and hoped in *chance,* the providence of those who have none.

Despite my endeavours to drive remorse away, the thought of what I had done did not cease to pursue me. My nights were restless or troubled with painful dreams; I could no longer indulge in reading or in walks, as before; my appetite, too, was lost. The tuitions to which I had fortunately to devote a considerable portion of the day, were alone able to afford me a little tranquillity, by temporarily removing the annoying idea from my mind.

An incident which I little anticipated caused the girl to be removed from the house, and led me to hope that she would not object to leave the town, where her presence was a permanent danger for me. She, either on purpose (as she told me), or otherwise, got drunk, and received her immediate discharge. It was in May, two months before her quarter was over. In the precipitation of her dismissal, I found only time to direct her to go home and to wait for me, on the next Sunday, at an appointed place, when I should see what best was to be done. The ladies of the house consented to accept, for the two remaining months, of the services of her sister, who was then out of employment. The girl had always assured me that no one had the slightest suspicion of her state. I was, therefore, not a little surprised and annoyed when I learned from the new comer that *she* had not made her pregnancy a secret with all her family.

At our first interview, I expressed to the girl my dissatisfaction at her imprudent disclosures, and, as the only remedy, my willingness to send her away to some distant place, until vacation, when it would be easy for me to take her to France, and to leave her with my family, whom I should inform of what had taken place, but without saying a word about her having been my servant. Had such a plan been put into execution, everything could still be repaired, or at least the impending danger was indefinitely removed. There might be vague rumours about her absence, but nothing more. I should have left off drinking whisky, in consequence of my mind being more at ease, and attended to my daily occupations with a new courage. Such is, at least, what I then intended to do. Unfortunately, my proposal was drily rejected; she would not go away; she was afraid I should leave her; she wanted to live in town, &c.; or she would make everything known.

I submitted in despair to her haughty wishes, and gave her money for lodgings. She hired a room in a retired part of the town, and came to live there, not alone, but with her mother and a niece, the two latter saddling themselves on my shoulders, as if one encumbrance were not sufficiently heavy. Demands of money succeeded each other with a fearful rapidity, so that I found myself quite unable, for want of cash, to take my usual trip to France.

At that period of the year (July), the harbour of Derry re ceived a number of French vessels, which gave me a daily opportunity of acting as interpreter between the merchants and the captains; but at the same time I neglected my private lessons, a fault which had never occurred before. Being a constant prey to sinister presentiments about the future, I used to drink wine and brandy on board, without, however, being ever sick (this fact I cannot account for); only, every morning when I got up, there was a kind of tremulousness in my limbs. I could scarcely take up a glass to my mouth without spilling a part of its contents; my walk was unsteady, and my speech broken, more difficult than usual, unless I got animated. The mind seemed to preserve its soundness; I had several times to draw up reports, which scarcely took more time than that of writing them down.

In this manner did I pass the month of July, be it said to my shame and deep regret. My visits to the girl were also frequent; it seemed as if an evil genius carried me there, though I well understood their danger and impropriety. I think that by that time I had lost a great deal of control over myself.

In August, the re-opening took place at Foyle College and at three other schools which I used to attend. The Rev. Mr. Henderson sent for me. I was not at home. Fortunately, a gentleman who also kept a school, and who was greatly attached to me, came on board an Italian ship, where he found me. He most justly said that he could not understand my way of living for the last month. There must be something wrong. That if I did not resume business immediately, he was afraid I should lose my pupils in town. He had been told something very painful to him, about my now taking to drink; but he did not believe that. He then carried me to his house for dinner. There he informed me that it was reported in town I had married my servant. This I denied.

My friend's lecture seemed to shake off my torpor for some time; I left off visiting vessels, to resume business.

Notwithstanding what had been rumoured, every one received me well. New pupils came to me, so that I could number upwards of fifty of them. But if this increase was gratifying to me, there were repeated calls on my purse which produced a very different effect. I continued to drink, and drank the more, on thinking of the fast-approaching time when there would be a living proof of my guilt.

This took place in November; as a rigorous consequence, I lost my situation at college and in another school. I did not repine. I acknowledged within myself that I deserved it. My remaining pupils were still in sufficient number to afford me the means of a livelihood. In order to avoid any further scandal, I

earnestly advised and prevailed on the girl to leave town. I rented for her a house in the country, about four miles from town. Had I thought that marriage would not have made things worse, I would certainly have married her, but out of all the persons to whom I spoke on the subject, Mr. Henderson alone gave me to understand that it was the only means of atonement from an honest man. It is true that when I asked him if my compliance with his advice would entitle me to a further attendance in his establishment, he answered that he could not employ me any longer, on account of the many respectable families whose children were at college, and who would object to the continuance of my tuition there.

Matters remained in this state until December 28th, when I went out to the country (as if led by my evil spirit). Hard drinking there for several days, joined to quarrels arising from constant demands of money, brought on me sickness and such exhaustion, that I could not leave my bed. From December 28th to January 13th, when I felt the real symptoms of the disease, I did not eat *one ounce of bread daily.* My only food was whisky, which I am sorry to say they were always ready to minister to me.

Until the 12th, I continued extremely weak, but felt so tired of the bed, that I got up.

Here, Monsieur le Docteur, I will endeavour to convey to you an exact idea of my disease in all its successive phases. I recollect everything so distinctly, that I can speak in the present tense, as if I were just *in the act of suffering.*

My night, as many others before, has been altogether sleepless. Itchings, hitherto unknown, are felt all over the body, and render my skin sometimes painfully tender, sometimes quite benumbed, as if it were dead. Diarrhœa increases my sufferings; I am so dizzy, that I cannot walk in the room without groping along. It seems to me as if there were small flies before my eyes; I hear their humming. If I look out through the window, all the objects assume confused, but not yet fantastic shapes. The itchings do not leave me; they are very troublesome, and make me worse. Cannot taste any food, and this day abstain from taking whisky. I retire to bed; no rest whatever; the itchings keep me in continual movements. Very early in the morning, and long before daybreak, I think I hear two or three peals of thunder, which frighten me very much. When I open my eyes, I see no more flies, but ignited small globules, like sparks. They are in myriads. I hear something like the ringing of many, many bells, and remark that if I rest my head on the pillow the din is really frightful. At times, I fancy that there are mice or rats running to and fro with their usual cries, under my head, inside the pillow. The day breaks in; I want to get up. My bed has become a bed of torture for me. I try to walk a little in the room, but weakness compels me to sit down. My food consists only of a few cups of tea, without any bread, for which I feel no taste. Several times in the course of this day, I have *des envies de vomir*, but I cannot. I look at the fire; the burning peat has asumed strange fantastic forms, which seem to be animated. As I cannot sleep, I sit up very late, in the hope that I shall, from mere exhaustion, enjoy a little rest. Now and then, I take a cup of tea. I feel well nowhere. Sitting is often replaced by two or three turns about the room. Whatever position I may take, weariness, discouragement, anxiety, press on me. I attend to a conversation whispered between the mother and the daughter. They seem to talk about me and my affairs. I several times fancy they utter the word France, and my name, accompanied with curses. I think they are alluding to the possibility of my returning alone to my native country, but they will not let it be done; they will prevent my departure. The old woman says that her daughter had better have drowned herself. I then recollect that she for the last days has been very moody, because my money (I imagined) was drawing to an end. Yesterday, I sent the son and brother of the two women for some money due to me, but he has brought back a cheque, which I alone can get cashed. I go on walking as if I did not listen. I am very far from being at ease, especially when I recal to mind that this is a lonely house, in a bleak, deserted part of the country, and that I should have to deal not only with the two women, but with the brother, a stout fellow, who has required no invitation to take up his abode with us, and who seems rather too much inclined to idleness. My apprehensions are, moreover, roused by the fact of my possessing the above-mentioned cheque. They might believe that they too can get it cashed at the Bank. At about twelve o'clock, I want to take another cup of tea with the two women, who are still up, and sitting near the fire. They prepare it; but I fancy I see the mother slip some black thing, like tobacco, to her daughter; I approach the fire; and again the mother tries to hand another lump of black stuff, but she drops it. I see the object of my suspicions lying on the ground; the mother tries to get it under her foot, which she stretches out in that direction, but she cannot succeed, and I suppose she is afraid I should notice her movements. The daughter looks uneasy. I am sitting between both of them, watching their motions in deep silence. At last, I avail myself of the first opportunity to pick up the obnoxious black lump, and I thrust it into my overcoat pocket. I am trembling from fear; I feel that I should hardly be able to speak. The sinister idea strikes me that they want to administer me poison, and the word vomica nux often presents itself to my mind. I get up from my seat, and resume my this time very unsteady walk, until the old woman presents me one of two bowls full of tea. I take it with a tremulous hand, and in a broken voice say to the daughter, "Drink it; I wish you to drink it;" but she would not; she does not want it. I then see my suspicions confirmed. I seize the two full bowls, and run with them out of the house, crying out: "You are two wretches; you wanted to poison me." I take the direction of the nearest house, in order to show the contents of the two bowls, but before reaching it, I let them fall, and pursue my way. I knock at the house, entreating that the door should be opened to me. A woman (the only grown-up person I see in the house) asks me what I want. In a most agitated tone I say that I have been nearly poisoned, and that I shall make an application to the magistrates about that. As I am speaking, the brother comes up. He has been awakened by the two others. Assures me that I am mistaken. "Well," said I, "come with me to any place where we may find a light, and I will show you that I am not mistaken. I have in my pocket an unquestionable proof of what your friends intended to do with me." "You are wrong, sir," replies he; "nobody wishes you harm; come you back to the house."

Fear prevents me from acceding to this request. I ran off through boggy grounds in the direction of a public-house, on Derry-road, about half a mile from the place. From the beginning of my flight I have lost my slippers, and have but a pair of stockings on. The night is very dark, and the rain is falling in torrents. I have to make my way through pools of water, dikes, rills, fences, and hedges. By day the task would be difficult, as there is but one very narrow and uneven path leading to the road—I do not keep the right direction for a long time; I hear close behind me the voices of the brother and sister; they are engaged in my pursuit. This idea increases my terrors. In the hope of escaping from that pursuit which I ascribe to bad motives, I leave the path and continue my run at random. I can assure that I am not less than half an hour wandering about, often stumbling in the marshes, often finding myself back again at places I just left a few minutes before. I once keep myself hidden in a ditch with water up to my knees; the voices are but a few yards behind me. Here is the road at last, but I see no public-house, and the darkness does not permit me to ascertain whether it is situated on my right or on my left. I take to the left, which is the wrong direction; I pursue my flight; the thought many times striking me that *God* has this time more obviously than ever saved me from an untimely grave. I pray along the road for the forgiveness of my past errors; I promise henceforth to behave like a true Christian, &c. . . I feel not only refreshed and encouraged by my prayers, but much stronger than I could have expected from the extreme weakness I felt on the preceding days. After half an hour at least of this run in the opposite direction to the pot-house, I begun to think that I must have found it, if I had taken the right way. I therefore retrace my steps, with unabated speed, determined to knock at every door and to speak out concerning my escape from the lonely house. Strange to say, out of at least five or six houses where I stopped, knocking repeatedly for several minutes and crying aloud for admission, I receive answer but from one. A man comes to the door without unbolting it, and rudely says the only words, *Cut away.* I am nowise disheartened.

On my arrival at the pot-house, I recommence rapping, and begging that they should be so kind as to open the door, for I am in great need of a shelter. A dog alone answers my knocks by his barking from inside. The fact is, that my pursuers got to the tavern before me, and there asking if anybody had called, said there was a man on the road, who was out of his senses, and who perhaps would ask for admission; the landlord had better not let him in. Such is the account given since to me by the girl who went to the house along with her brother, and obtained admittance on pretence that they wanted candles. The landlord, being warned, does not move from his bed, and lets me stand out until I perceive that he has been prepared for my visit. I then make up my mind to return to Derry, where I should inform the police of what, in my fancy, has taken place. Indeed, I have not the least doubt but a criminal attempt has been made against my life. Curiosity, however, soon altered my resolution. When I reach a place on the road where a lane branches off in the direction of the lonely house, an unconquerable desire bids me go and see from outside the window what is passing in there. As if I foresaw some bad encounter, I break from a hedge a short stick which is to be my weapon, in case of danger. I have not proceeded many yards in my new direction, when I am stopped by two men carrying sticks. Who are they? The brother, and a fellow of his acquaintance who is known under the name of the *dummy* (he was dumb). The former imperiously invites me to return to the house, where no harm is intended against me. I feel so frightened that, to show I do not wish to make any disclosures about the events of the night, I throw the black stuff out of my pocket and, though reluctantly, follow the two men.

When I come in, I find there the woman to whom I first applied. She appears to be on good terms with the others, and I learn that the dummy is her son. This raises my suspicions about her. She endeavours to make me understand that I am quite mistaken about what I call *poison*, it was nothing but *soda*. How far this assertion is true, I cannot say; but cannot help thinking that *soda* is not black.

They make me sit down and change my clothes, which are dripping wet. The brother goes out for some whisky, which, they say, will do me good. On his return from the public-house, I take a small glass mixed with water, taking previously care that it should be tasted by the others. Contrary to my expectation, I do not feel weary at all. I look at my feet and hands which, to my great wonder, bear not one single mark of a scratch, although I have been running for two full hours, shoeless, treading on sharp stones, and often obliged to jump over ditches or to force my way through thick thorn-hedges.

This I consider as the greatest proof that I was guided and protected by some supernatural Being. I say so to the people, but I am by no means reassured in mind. I reflect that I am in a sinful state, without any hope of forgiveness, were I to appear now before the Supreme Judge. My fears increase in proportion as the others endeavour to prevent my escape. I fancy they are all decided to make away with my life. I entreat them to let me go; I confess that I am afraid of them, &c. Strange visions throw my mind into great excitement; every object takes a hideous shape and moves about. I look at the windows; diabolic faces are laughing at me. Their laughter makes me shudder. On whichever side I may turn, a chilling wind is hissing by my ears, with unearthly shadows passing before my eyes. If I look towards the door it is opening noiselessly, and I imagine I see somebody whose terrific head is peeping in. I start painfully at the least noise and utter lamentable cries. This lasts for hours, while I am sitting by the fire.

I am prevailed upon to retire to bed. Do not feel any better. Vainly do I shut my eyes in the hope of avoiding the sight of everything; horrid phantoms appear amidst the darkness. I feel as if I were pricked behind with a sharp instrument. The itchings are insupportable. I am a prey to continual restlessness, mixed now and then with the cries produced by an unexpected noise, such as the fall of a chair, or by new visions.

At the break of day the excitement subsides a little, and gives place to a fainting fit of short duration. For some time no new starts occur; but the confused ringing of bells continues; my sight grows very dim; I see nothing but monsters calculated to keep up my fright. Starts soon return more painful; even one of them throws me down on my knees, compelling me, as it were, to address a fervent prayer to our Lord for the pardon of my past life.

From this day (13th) to the 27th no amelioration in my state. I look on the house as a cursed place and remove to Derry, again followed for my misfortune, by that family whom I dread, in spite of all reasonings. As if their number were not sufficient, the sister had also made herself at home. I say repeatedly that I don't want their presence, that there is but one whom I ought to provide for; they stick to me like harpies, and take no notice of my remonstrations. They most likely will not go so long as there is anything to eat.

Driven to desperation, if I take no food, I keep on drinking whisky, not so copiously as before, but yet a great deal too much. I wonder how eagerly they give it to me, and advise me to take another drop whenever I complain of my extreme weakness. On my arrival in Derry, new fits of faintness: I sent for the priest, in order to receive his consolations; for I do not expect to live much longer. The reverend gentleman who has come to see me, perceives at once that medical assistance is to be had immediately. He therefore leaves me, and shortly after returns with a doctor, to whom I explain what I can about my complaint. The women are upbraided for having given me so much strong drink in my present state. The two gentlemen advise me to leave the place and the company, and to come *alone* with them. They take me to a respectable hotel, where they get a comfortable room for me; a nurse is also engaged to sit up all night in case of need.

Despite the excellent accommodation I have now obtained, I cannot enjoy one moment's rest. Besides my other sufferings, a new one came to complicate the symptoms of my disease. It is the fancy that I hear every one in the hotel speaking ill of me, and even the dreaded family is here too. They all proffer alarming threats; they want to have my life. It is wonderful how faithfully their voices are reproduced. I would swear that mother, daughters, niece, brother, and even the infant are below stairs in the kitchen. I cannot be undeceived by the kind words of the landlady. I am even so foolish as to believe that she has given them admittance, contrary to the orders of the doctor. The night-nurse does not escape my distrust either. In short, I see but the face, I hear nothing but the voices of those which, from want of other words, I shall call my persecutors. They are here, now in the kitchen exciting their hearers against me, now outside the door, in the street. Cries distressing for me, such as, *Stop, stop the mad dog,* often fall on my ears and cause me to spring out of the bed, and to look out either in the stairs or in the street. Such has been my daily state during the time that I stopped at the hotel. Meanwhile I received frequent visits from the priest and the doctor; my conversation with them was always sound, so far as the girl's family was not alluded to; for in the latter case I could not believe that I was the sport of a delirious imagination. Laudanum was several times administered to me in large doses, but to no purpose; on the contrary, I am of opinion that it did me more harm than good, for I then used to see everything more confusedly, and as if dancing before me. Unnecessary to say that appetite did not return, I had only some refreshing drinks prescribed by the medical gentleman.

Reasons of economy, and the advice of the doctor, induce me to go to the infirmary. I am conducted there by the doctor himself, and I obtain a bed in a small quiet ward generally used as a room for surgical operations. There are two other patients opposite myself, and the cook sleeps in a fourth bed on my left. Although restless and unable to sleep, I have no starts and make no noise whatever for many hours. It is two o'clock A.M., I am wide awake. I look towards the bed on my left and I have this painful vision.

I am (in imagination) in the lonely house. Sleep has overcome me. The mother lies in the other bed, on which my eyes are fixed, with the little niece who says: Grannie, where is Mr. D.?

*Grandmother.* He is away; let me alone.

*Child.* Grannie, where is Mr. D.?

*Grandmother.* Hold your tongue, he is killed, killed dead.

This lasts for several minutes, being repeated many times. All of a sudden I hear the mother ask the girl lying beside me: Does he sleep?

*Girl.* Yes.

*Mother.* Well, make haste off then, have done with him. It is two o'clock; we shall have time to run away.

*Girl.* I cannot find that cursed knife. Ah! here it is, I have got it . . . . . .

Then I feel twice something like a pointed knife penetrating into my back. I utter a feeble cry, then all is silent. The mother again says: Well, have you done?

*Girl.* Yes; he has enough. Let us get off.

And it seems as if the mother were leaving her bed, and the girl slipping cautiously from mine. At the same time I hear from outside the voices of the sister and brother, who say: Quick, or we shall be caught. . . . .

They all escape, and immediately after two doctors come to examine my body, which I fancy is lying inanimate in an adjoining room. One of them says in French: *Il est mort, il est bien mort.* The other also, in French: *Le pouls bat-il encore? Voyons. Oui. Alors il n'est pas mort. Non, non, il n'est pas mort.*

They carry me away, and another scene offers itself to my eyes.

The mother and my ex-servant are gone; they are superseded on the tragic theatre by the sister and brother; the latter leading the little girl by the arm, and the former holding the infant. She is looking for the knife used against me. She finds it on the edge of a small well opposite the door. It was to be thrown into the water, but in the precipitation of her flight, my servant has missed her aim. I see (for you have not forgotten, Monsieur le Docteur, that my eyes are wide open) the sister stab the poor infant, and, to stifle his cries, she, with a curse, tears his tongue off and throws him into the well. The little girl is also got rid of because she cries that they have killed Mr. D. and her cousin. I am so well awakened that I relate to the night-nurse particulars after particulars, as they are taking place. My sense of hearing acquires, on this night, such a degree of quickness that I hear every quarter of an hour striking by the town-clock, and every time I say, It is half-past two, it is a quarter to three, &c. . . . . For me there are very audible sounds which are hardly perceptible by others.

A few minutes after, when I think they have all escaped, here comes the dummy's mother to fetch water; she discovers the infant's body, and cries out, *Murder!* The sister makes her appearance again. The woman accuses her of murder; a struggle ensues, the result of which is that the woman is strangled.

Then I hear a confused noise produced by voices and the sound of heavy steps. It is the police. They have arrested the murderers, and bring them back. I see every one of them. There is the mother, there are the three others, handcuffed, and closely watched by the officers, who are armed with carbines, and have received the order of firing, should the prisoners attempt to escape. Now, too, the body of the woman is discovered, and I hear several voices say: This is really a cursed place: the house of murder.

Again the scene changes. I feel some one in my bed, who speaks to me. He says that he is my good genius; he has come to protect me from the wicked; but I must be truly repentant. I therefore pray for a long time in a low voice, until I fall asleep from exhaustion. My slumber is very short and agitated. I awake before daybreak. Now the scene of the night is continued. I hear criers in the street announce that the family ——, convicted of murder on the persons of ——, have been sentenced to death, and are to be executed on the same day. It seems to me that I am under a strange sky. The fog is very thick. I hear nothing but the cries of sinister animals, such as wolves, dogs, and the shrieks of geese, the croaking of frogs, mixed with the monotonous voice of the criers. I again fall into unconsciousness until it is light. I have been very restless, but not so noisy as to prevent the other patients from sleeping. The nurse alone knows what my imagination has seen.

On awaking, my eyes wander from one object to another, and remain fixed on many pieces of wood, used by the doctors in their surgical operations, and which lie topsy-turvy on a press in a corner of the room. I first shrink from the sight, for now the top of the press is occupied by living beings: here are the mother and my servant again; then, on their rear, the sister and the brother. But in what state? My good genius tells me that such is the visitation of God on great criminals. The mother has a cadaverous face; her eyes are sightless and white; her hair has assumed the colour of flax; the rest of her body is concealed from me. The daughter is closer to me; she is dressed as for a *fête*, but her head is nearly bald; the hair has fallen off in the space of a few hours. There is a large stain of blood impressed on her brow, and a candle (like a sepulchral lamp) is burning beside her. They both stare at me now and then, like people who look but do not see. The two others, sitting exactly behind, present a disgusting aspect. The sister is as pale as a corpse; her hair, too, is white, and very thin on the forehead; the lips emit a kind of sanguinolent foam; the head performs the oscillations of a pendulum; she is an idiot. The brother's appearance is that of a hideous cripple; the head has decreased nearly to nothing, and would scarcely be visible, were it not for two green eyes, obstinately fixed on me, but without any significance. He reminds me of what I have read about *cretinism.* I forgot to say that there is a fifth actor in this tragic tableau—the young girl, with curling hair, neatly clothed, leaning sometimes on her grandmother, sometimes on her aunt, and repeating at intervals: Grannie, or Auntie, where is Mr. D.? to which question the only answer given is: Hold your tongue; he is away, he is dead, killed dead.

This spectacle keeps my mind in excitement for the whole day. Visitors come in and look with wonder on those strange beings, from whom my eyes cannot be removed. Those visitors say, It is strange—very strange indeed! In order to escape from the frightful sight, I once run off the room. The doctor, who happens to be in the next ward, brings me back, but cannot persuade me that I am mistaken. On another time, I fancy that an iron bar, placed to support a curtain above my feet, pours on me something whitish, like melted lead, which burns all my body. The same imaginary tube is sometimes turned against the family, and seems to produce on them the same effect as on myself. Again, I think I hear the voice of a gentleman, the head man of the committee, who visited the wards a few hours ago. He is upbraiding the doctor, in most unbecoming terms, for having given me admittance, while there are so many poor Irish dying out for want of medical cares and of bread. A quarrel and a fight ensue, the result of which is, that the doctor is shot dead. I hear the report of the pistol and the cries of many persons calling for the police, who, after much delay, arrive and capture the murderer. Before the arrival of the police, I once imagine that he is ascending the stairs to kill me; I jump from my bed, and conceal myself under another. I am dragged from under it by a day-nurse; then I run off again, at the risk of killing myself in rolling down the stairs. I am caught at the bottom. They carry me up again to the room, not without an obstinate struggle on my part; for I am afraid of new visions. The strait waistcoat is resorted to. They fasten me so tight that I can no longer move: my breathing is even greatly impeded by a leather strap pressing on my chest. Night has come; I begin to utter cries of distress, because I see the unavoidable figures from the press quit their immobility and join in infernal fits of laughter.

Exhaustion again delivers me from consciousness. I am aroused from my torpor by the endeavours of the attendants to make me swallow some medicine. The idea immediately strikes me that the potion forced into my mouth is poison, and I spit it out. No more rest during the night. My eyes emit sparks of fire which fill the room. My persecutors are still there; no longer on the press, however, except the brother, who has resumed his natural form, and seems ready to spring on me. They are lying in the other beds; there seems to me as if an electric thread were carrying to them my inmost thoughts, which they repeat aloud. On the other hand, I can get, through the same imaginary thread, a knowledge of their designs against me.

My good genius has not left me; he bids me look for strength in a sincere prayer, and pours on my enemies the same white fluid already mentioned. It is directed from my side to the places they occupy, and instantly reduces them to silence. From time to time, also, when I pray without fervour, or when I entertain any doubt about my good genius' power, the shower is turned against me, from the iron bar, and especially directed to my head. This has the effect of fire; it burns my body all over so sorely that I cannot help crying.

The heat is oppressive; the room is full of a reddish smoke, at intervals chased out, through the door, by a blast of wind. I tell the nurse that, although the door is opened, I am afraid we will soon be blown up, if she does not put out the gas; she answers that there is no occasion for it, as we are in no danger, and I had better sleep, as if sleep were to come at my command. In my restlessness, I fancy that there is the head of a wolf, with glaring eyes, on the bolster; I pray for a long time; the head disappears. I am a little refreshed, but cannot sleep. My mind soon turns to other fantastic thoughts. I am no longer an inmate of the infirmary. I am kept a prisoner by my persecutors in a small house, where they endeavour to smother me by shutting the door and lighting a fire of straw in the middle of the room. The mother and sister are more implacable than the others, and appear to enjoy my torments. Whilst I am a prey to great sufferings, and scarcely able to breathe, I hear from the street a voice which I immediately know to be the voice of my brother-in-law. I wonder that he has come from Paris to Ireland. He answers that he has come with my sister for the purpose of settling as a French teacher. I turn then his attention to my present miserable state. I implore his assistance; I entreat him, in the name of my sister and of our former friendship, to deliver me; but he laughs at my supplications, and even joins with my persecutors, whom he also excites to show no mercy and to take no heed of my cries, as there is nobody at hand to hear. I hear him walking up and down the street; he is with my sister; they both say, repeatedly—*Il est perdu, il n'est pas perdu. Eh bien! Oui, il est perdu. Tant pis pour lui.*

At daybreak the visions disappear for a little time. My lips are parched from crying; my feet are now cold. I complain to the nurse. They give me a drink of milk, and place a jar of hot water at my feet. I remain thus quiet, and as if prostrated, until the doctor comes in on his round. He inquires of my state; feels my pulse; asks if I could sleep last night. He is told that I was noisy, speaking about dangers, praying aloud, &c., and that I would take no laudanum. He kindly remonstrates with me, saying that everything is prescribed for my good. (*That medical gentleman was well known to me, and he also knew me very well, as I used to give lessons in French to his family.*) Unfortunately the subordinates have a rough way of discharging their duty. They, in my helplessness, illtreat me, threaten me now with a stick, now with the red-hot poker, which they approach to my mouth. In these illtreatments and menaces my delirious imagination sees nothing but a continuation of the tortures inflicted on me by my enemies. I look upon the night-nurse, the day-nurse, and especially on the *infirmier*, as people under the power of Satan, whom my prayers alone can drive away.

Their drugs, too, I consider as being made by an evil hand, and only calculated to soil my soul. I have made up my mind to accept of nothing, except water or milk.

In the course of the day, I come to think that my mother is dead, and that my eldest sister has arrived, and wants to see me. She stops with my brother-in-law and my other sister; but she cannot obtain any information about the place where I am kept. My persecutors re-appear; I find myself in another house quite unknown to me. Besides the family, there are strange faces, equally hostile. They want me to sign a promise of forty pounds, in return for my release. I consent to their request, but when the signature is given, they wont let me go; they now say that they must have their revenge. I am stretched on a mattress, tightly fastened with ropes and leather straps. I can hardly move my head. Presently my legs are stripped, and the toes of my feet covered over with a thick layer of fat meat. What do they intend to do? From their conversation I at last learn that my toes are to be devoured, along with the meat, by a huge dog of theirs, whom they have taken care to keep in good appetite for the *occasion*. The dog cannot be got for some time, during which I am a prey to frightful apprehensions. He is brought in by two men, and rushes, from the first, upon my feet, which he dreadfully mutilates. I hear the cracking of the bones under his teeth; I again cry and weep pitifully. There are many people—men and women—around me. They all seem to enjoy the spectacle, and take no notice whatever of my cries and tears. I have lost all remembrance of what followed; I suppose that I fainted. The fit, however, was short; for at night I have the following dream. (Let it be understood that all my dreams are nothing but visions; for they take place when I am wide awake, and when my eyes are open; my properly styled dreams have left no recollections in my mind.)

(*To be continued.*)

---

